# The Psychological Consequences of the COVID-19 Pandemic in Adults Treated for Childhood Cancer

**DOI:** 10.3390/curroncol29060327

**Published:** 2022-06-06

**Authors:** Asmaa Janah, Nadia Haddy, Charlotte Demoor-Goldschmidt, Nicolas Bougas, Jacqueline Clavel, Claire Poulalhon, Brigitte Lacour, Vincent Souchard, Angela Jackson, Leonie Casagranda, Claire Berger, Rodrigue Allodji, Chiraz El Fayech, Brice Fresneau, Florent De Vathaire, Agnes Dumas

**Affiliations:** 1 Université Paris Cité, INSERM, ECEVE, F-75010 Paris, France; asmaa.janah@inserm.fr (A.J.); nicolas.bougas@inserm.fr (N.B.); agnes.dumas@inserm.fr (A.D.); 2 University of Paris-Saclay, F-94800 Villejuif, France; nadia.haddy@gustaveroussy.fr (N.H.); c.demoor@hotmail.fr (C.D.-G.); vincent.souchard@gustaveroussy.fr (V.S.); angela.jackson@inserm.fr (A.J.); rodrigue.allodji@gustaveroussy.fr (R.A.); 3 Gustave Roussy, Department of Clinical Research, F-94800 Villejuif, France; chiraz.fayech@gustaveroussy.fr (C.E.F.); brice.fresneau@gustaveroussy.fr (B.F.); 4 INSERM U 1018, CESP, Radiation Epidemiology Team, F-94800 Villejuif, France; 5 CHU Angers, Paediatric Oncology Department, F-49100 Angers, France; 6 François Baclesse Centre, Radiotherapy Department, F-14000 Caen, France; 7 Centre of Research in Epidemiology and Statistics, INSERM, F-94800 Villejuif, France; jacqueline.clavel@inserm.fr (J.C.); claire.poulalhon@inserm.fr (C.P.); brigitte.lacour@univ-lorraine.fr (B.L.); 8 National Registry of Childhood Cancer, Paul Brousse Hospital (AP-HP), F-94800 Villejuif, France; 9 Regional University Hospital Centre of Nancy (CHRU Nancy), F-54511 Vandœuvre-lès-Nancy, France; 10 CHU St Etienne, Paediatric Oncology Department, F-42055 St Etienne, France; leonie.casagranda@chu-st-etienne.fr (L.C.); claire.berger@chu-st-etienne.fr (C.B.); 11 University of Lyon, University of Jean Monnet, INSERM, U 1059, F-42100 Saint-Étienne, France; 12 Gustave Roussy, Department of Children and Adolescents Oncology, F-94805 Villejuif, France

**Keywords:** childhood cancer survivors, consequences, COVID-19, lockdown, mental health, psychosocial, survivorship

## Abstract

Background: Compared with the general population, childhood cancer survivors (CCS) could be at greater risk of psychological distress following the emergence of the COVID-19 pandemic. Purpose: This cross-sectional study assessed the psychological consequences of COVID-19 on the mental health of CCS. Design and participants: In December 2020, we interviewed through an online self-report questionnaire, 580 5-year CCS participating in the French Childhood Cancer Survivor Study (FCCSS) cohort. Methods: We first compared the mental health score of CCS with that observed in the French general population of the same age and gender. Subsequently, we studied predictors of the mental health score of CCS. Results: External comparisons revealed that the mental health score of CCS was similar to that of the general population. Among CCS, almost 42% stated that their psychological state had been worse during the lockdown. Predictors of poorer mental health included, among others, female gender, reporting a change in the occupational situation, having a relative who had been hospitalized or had died following COVID-19, and a greater perceived infection risk. Interpretation and Implications: Given the pre-existing vulnerability of some CCS to mental distress, the additional psychological consequences of COVID-19 in vulnerable survivors should receive attention from health care providers.

## 1. Background

Caused by the severe acute respiratory syndrome coronavirus (SARS-CoV-2), the 2019 coronavirus disease (COVID-19) emerged worldwide and was declared by the World Health Organization (WHO) a global pandemic in March 2020 [1]. By April 2020, half of the world’s population was in some form of lockdown, with over 3.9 billion people in more than 90 countries or territories being ordered to stay at home and to minimize non-essential contact during what has been called “the great lockdown” [2,3] and causing health, social, economic, and psychological issues for all [4,5].

Several studies conducted in the general population have shown that COVID-19 has caused significant psychological distress in many people [4,6]. In France, an online survey carried out after the initiation of the great lockdown (i.e., from March to May 2020) showed that a third of the participants presented psychological distress during this lockdown, affecting more frequently women and people with a poorer financial situation [4]. Indeed, the implementation of this lockdown, added to the uncertainty surrounding the pandemic and the transmission modes of the virus, increased the level of anxiety and psychological distress [7].

Moreover, people with comorbid chronic diseases are at higher risk of a severe course of COVID-19 and death [8]. Because of their previous cancer diagnosis and treatment, childhood cancer survivors (CCS) are likely to experience the chronic conditions associated with a severe course of the disease [9,10]. In addition, fear of the disease could lead to forgoing care, which may amplify the psychological concerns of CCS needing care during the pandemic [11,12].

One previous study conducted before the pandemic showed a prevalence of psychological distress in long-term CCS approaching the clinically significant levels seen in the general population [13], while other studies showed greater distress in these survivors compared with the general population [14] or compared with their siblings [15]. However, scientific data on the psychological health of long-term CCS in the COVID-19 era are very scarce. Therefore, we sought through the present study to assess the psychological consequences of the COVID-19 pandemic in CCS and to identify its predictors. 

## 2. Methods

### 2.1. Study Population

We carried out a cross-sectional study among the participants of the French Childhood Cancer Survivor Study (FCCSS) and compared the collected data to those of the French General Population (FGP) (i.e., reference data).

### 2.2. French Childhood Cancer Survivors Cohort

This cohort is meant to assess the long-term effects that may have a negative impact on children and adolescents treated for cancer [16]. It includes 5-year CCS diagnosed between 1945 and 2000 with a solid tumor or a lymphoma and treated before reaching age 18, in five centers in France. The cohort currently includes 7670 five-year survivors, of which 5023 were alive and with a known postal address since 2005, the year when a baseline questionnaire started to be sent to survivors. Of them, 3293 answered this baseline questionnaire. In December 2020, a new online self-report questionnaire was implemented on REDCap^®^ and sent to 2728 survivors who had provided their email address to be contacted for additional surveys after completing the baseline questionnaire. 

The FCCSS protocol has been approved by the INSERM national ethics committee and the French National Agency regulating Data Protection (CNIL No 902287). Consent was obtained from patients, parents, or guardians according to national research ethics requirements.

### 2.3. General Population

Reference data on mental health were extracted from the COCONEL survey, which is a nationally representative survey carried out in the FGP during and after the great lockdown, in spring 2020. It used a quota sampling method to ensure representativeness of the French population and assessed risk perceptions toward the pandemic [4].

### 2.4. Data Sources

Self-report data from the online questionnaire were complemented by: (1) data from the baseline self-report questionnaire sent previously to the cohort participants, (2) medical records, and (3) medico-administrative data collected from the French national health insurance database.

### 2.5. Outcome Measures

The psychological consequences of the pandemic and the lockdown on respondents’ health were measured in the present study by using the Mental Health Inventory-5 (MHI-5) scale of the validated French version of the 36-Item Short Form Health Survey (SF-36) assessing health-related quality of life (HRQoL) [17]. The MHI-5 scale is a brief screening questionnaire measuring general mental health and comprises five items for detecting psychopathological disorders: nervousness, sadness, peacefulness, mood, and happiness [18]. A higher MHI-5 score reflects better mental health. The outcome measure was similar between the questionnaires used in cancer survivors and in the general population to obtain comparable data, both surveys assessing retrospectively the MHI with respect to the same period (i.e., the great lockdown).

In addition, CCS were asked to report whether their psychological health was worse, better, or the same as in the months leading up to the great lockdown: “Do you feel that your psychological health has changed during the great lockdown period?” No reference data were available for this outcome. 

### 2.6. Covariates

#### 2.6.1. Demographic and Clinical and Data

Demographic predictors included age at the survey (<39, 40–49, and ≥50 years) and gender. Clinical predictors were childhood cancer type (classified according to the International Classification of Childhood Cancer), primary cancer treatments (radiotherapy, chemotherapy), and chronic health conditions including cardiovascular disorders, diabetes, and second malignancies.

Demographic information (i.e., gender, date of birth, and date of diagnosis), tumor characteristics, and cancer treatments were extracted from medical records in the center in which the participants were treated for childhood cancer. Chronic health conditions, including cardiovascular disorders, diabetes, and second malignancies, were ascertained from physicians’ reports and were complemented by data from the national health insurance database [19]. Cardiovascular disorders included myocardial infarction, angina, heart failure, valvular diseases, cardiac arrhythmia, conduction disorder, and pericardial disease and were graded according to the Common Terminology Criteria for Adverse Events. Classification as grade 1 meant asymptomatic; grade 2 meant symptomatic but mild enough to remain untreated; and grade ≥ 3 meant symptomatic and treated, life-threatening, or having led to death [20]. Cardiac diseases were entered in the model as a binary variable without distinction of the grade.

#### 2.6.2. Socioeconomic Data

Socioeconomic predictors included education level, which was extracted from the baseline questionnaire and defined as the highest diploma obtained (below high school, high school graduate, or college graduate [bachelor or higher]). In addition, socioeconomic data were collected by using the online questionnaire and included possible changes in the respondent’s financial and professional status since the start of the great lockdown (i.e., reporting loss of income or job during the lockdown/since the start of the pandemic, yes or no). In addition to these professional indicators, the online questionnaire collected information on the living conditions of survivors during the lockdown (i.e., number of rooms, number of people living in the same accommodation, presence of a garden).

#### 2.6.3. Data on Risk Perception

The online questionnaire also collected the perceived infection risk of CCS by using a scale from 0 to 10: “On a scale of 0 to 10, how much have you been worried about the possibility of catching Coronavirus?” Besides, the participants were asked to report if they feared that their health might be endangered by the working conditions due to the COVID-19 pandemic.

#### 2.6.4. Data on Health Care Use

Health care use was assessed by asking the participants to report whether they had forgone or postponed one or more medical appointments: “Since the start of the pandemic, have you postponed or forgone one or more medical appointments for yourself?”.

#### 2.6.5. Data on the Perceived Experience of the Pandemic

The participants were asked if the COVID-19 pandemic had led them to remember their childhood cancer during the lockdown: “Did you think more about your known illness as a child during the lockdown period?” Finally, the general experience and the consequences of both the pandemic and the lockdown were assessed by using an open-ended question. The answers were recoded into 13 categories, and quotes were extracted to give examples of the categories.

### 2.7. Statistical Methods

First, the demographic, socioeconomic, and clinical characteristics of CCS who participated in this study were described and then compared with those of non-respondents by using chi-square tests. Because some demographic and clinical characteristics differed between respondents and non- respondents, all statistics were weighted to account for the potential non-response bias [21]. The applied weighting coefficients were computed as the inverse of the probability of participating in the survey and then normalized. These participation probabilities (i.e., propensity scores) were calculated using a logit model as a function of age at the survey, gender, type of childhood cancer, and level of education.

Then, to compare the mental health status of CCS to that of the general population, we computed Z-scores and their 95% confidence intervals (CI) for the MHI-5 score (i.e., the number of standard deviations [SD] compared with the average estimate in the general population sample) adjusted for gender and 5-year age strata (25–29, 30–34, 35–39, 40–44, 45–49, 50–54, and 55–59). The standardized MHI-5 score was computed according to the following formula: Z = [(x − μ)/σ], in which Z is the standardized score, x is the raw score observed in CCS, and μ and σ are, respectively, the mean and the SD in the general population of the same age and gender [22]. This comparison was limited to those aged 25–59, that is to say, professionally active survivors. The smaller the absolute value of the Z-score, the closer the MHI-5 score of CCS is to the values of the FGP. A mean Z-score significantly different from 0 meant that the MHI-5 score was different between CCS and the general population.

Subsequently, univariate and multivariate linear regression was performed to identify predictors of the MHI-5 score. The results are expressed as β with their 95% CI.

All analyses were conducted by using SAS 9.4 software (SAS Institute Inc., Cary, NC, USA). Selection of variables was carried out by a stepwise procedure (significance levels for entering an effect into the model, 0.2; staying in the multivariable model, 0.05).

## 3. Results

### 3.1. Baseline Characteristics

Overall, from December 2020 to January 2021, 580 CCS responded to the online questionnaire, with a participation rate of 21%, after two reminders. We found an average time of 10 years between participation in the baseline questionnaire of the FCCSS and this survey.

The respondents were older than the non-respondents and were more likely to be college graduates, to have been treated with radiotherapy, and to have chronic health conditions (i.e., cardiovascular disorders and diabetes). The mean age at the survey (SD) of all respondents was 43 (10) years. Of all participants, almost a quarter were aged ≥ 50 years, 53% were female, and 59% had an education level higher than high school. Among the participants, lymphoma and nephroblastoma were the most common diagnoses, while brain cancer was the least common diagnosis (Table 1). Of all participants, 19% said their financial situation had deteriorated during the spring 2020 lockdown, 2% reported a termination of their contract by the employer, and almost 5% a stop or a significant decrease in their professional activity while they were self-employed. 

### 3.2. Comparison of the MHI-5 Score of Respondents with the FGP

Respondents aged from 25 to 59 years (*n* = 509) reported a mean (SD) MHI-5 score of 49.8 (10.0). Compared with the FGP, the overall MHI-5 mean Z-score (95% CI) was −0.01 (−0.10 to 0.08), showing the absence of a significant difference in the reported psychological status of CCS compared with that observed in the FGP. Besides, stratified descriptive analyses by gender, educational level, second cancer, or cardiac disease showed similar results, with no significant difference in the reported psychological status of CCS compared with that of the FGP (Table 2).

### 3.3. Predictors of the MHI-5 Score

**Figure 1:**
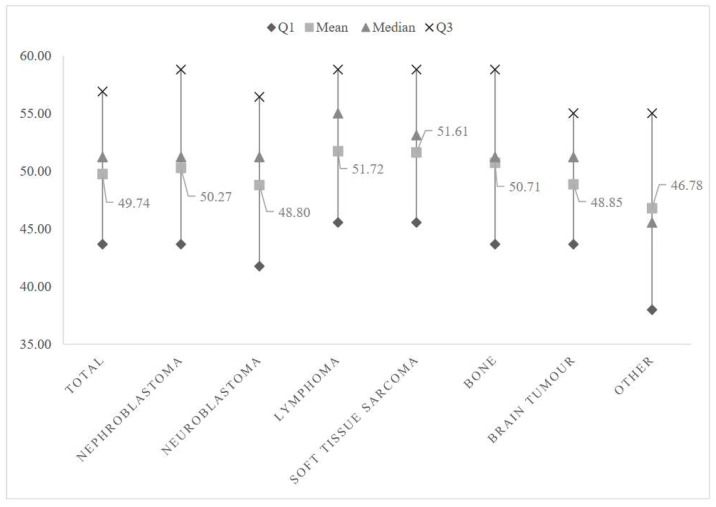
Mental Health Inventory-5 (MHI-5) score by cancer type.

Univariate analysis of the MHI-5 score by cancer type is presented in Figure 1; it shows an overall significant difference between cancer types regarding the MHI-5 score (*p* = 0.003). We observed a higher score in CCS diagnosed with lymphoma or soft tissue sarcoma, while those with a brain tumor reported a lower mental health score. In the multivariate analysis (Table 3), after adjustment for individual and clinical characteristics, predictors of poorer mental health included: female gender (adjusted β [aβ] −3.55, 95% CI −5.20 to −1.89), having a relative who had been hospitalized or who had died from COVID-19 (aβ −2.70, 95% CI −4.81 to −0.58), having lost his/her job at the employer’s initiative during the pandemic (aβ −7.64, 95% CI −12.87 to −2.42), being a self-employed person who had to stop or severely reduce his/her activity during the lockdown (aβ −7.50, 95% CI −11.13 to −3.86), remembering his/her childhood cancer more during the lockdown (aβ −2.45, 95% CI −4.20 to −0.70), and reporting a greater perceived infection risk (aβ −0.68, 95% CI −0.98 to −0.37). By contrast, participants living in a house with a garden (aβ 2.58, 95% CI 0.55 to 4.61) were more likely to report better mental health in the present survey. No significant association was found between the mental health score and chronic health conditions such as cardiac disease or a second malignancy. In particular, we did not find any significant results in sensitivity analyses where the cardiac disease was coded accordingly to its severity (i.e., grade).

### 3.4. Reported Consequences of the Great Lockdown and the COVID-19 Pandemic

Asked about a possible change in their psychological health, 42%, 10%, and 48% of all respondents said their psychological state was, respectively, worse, better, or remained the same during the great lockdown compared with the months before the start of the COVID-19 pandemic.

Overall, 38% of CCS wrote a detailed comment on their perceived consequences of the great lockdown and the pandemic in general. The most reported health-related difficulties by the CCS included an increased perceived infection risk and the inability to practice sport, which could worsen possible joint pain and musculoskeletal disorders. Besides, CCS reported difficulties in accessing supportive care or physiotherapy and difficulties in traveling abroad for in vitro fertilization appointments, for example. CCS also reported difficulties attending long-term follow-up medical appointments on their own. One survivor reported: “That day I went to my neurosurgery and dermatology consultations to follow up meningiomas and carcinomas, alone in the face of medical announcements (new carcinomas to be operated on) without my wife who could not support me as usual, since relatives were not allowed to go into the hospital. I will go alone on Thursday to my emergency appointment to see the maxillofacial surgeon and, maybe, to be operated. It’s hard!!!”

Some felt that their experience of childhood cancer had made them stronger to face the pandemic (*n* = 2). “Strangely, having known the disease in the past, we have a feeling of super power as if nothing could happen to us worse than what we have experienced. However, we are more worried about our loved ones.” Others highlighted the idea that life was short, a notion they had developed after their childhood cancer experience: “It reassured me in the idea that life is short, fragile, that you have to live it from day to day without knowing what will happen the next day, an idea that I had acquired since my cancer, as a child.” One last interesting comment came from a CCS who reported that the similarity between their childhood cancer and the great lockdown manifested in a feeling of deprivation of one’s own freedom: “The link that could be established between past illness and the spring 2020 lockdown period would be deprivation of liberty.”

## 4. Discussion

This study is one of the first to assess the psychological consequences of the COVID-19 pandemic and the resulting great lockdown in CCS. We found that more than 4 in 10 survivors said their psychological health had worsened during the great lockdown. Preliminary results from a study conducted by Forbes and colleagues in the United States appear to show a similar proportion (i.e., 42%) [11]. These results are consistent with previous evidence showing the significant psychological consequences of the global COVID-19 pandemic and the resulting restrictive measures [4,6,23]. At the same time and unexpectedly, we reported similar mental health among survivors and their general population peers of the same age and gender. This finding extends evidence reported by a study showing similar mental health in survivors of adult cancer during the COVID-19 pandemic and in adults without a cancer history [24]. Other divergent results have been reported by a study assessing the psychological distress during the COVID-19 pandemic in survivors of adult cancer and healthy controls, showing that cancer survivors reported a greater catastrophizing attitude in relation to the COVID-19 pandemic, while showing less psychological distress [25]. Conversely, this finding was not supported by another study reporting more mental health symptoms in adult cancer survivors compared with healthy controls [26]. The above-mentioned studies including control groups were conducted among patients who had cancer in adulthood. Indeed, there may be significant differences in the psychological consequences reported by this population and by CCS. Strict comparisons with these studies may be limited and should therefore be interpreted with caution.

The international literature has shown that long-term and sometimes short-term CCS have comparable or even better mental health compared with their peers in the general population [13,27,28]. Indeed, this statement concerns less physical health than mental health, in which the notions of psychological adjustment, posttraumatic growth, and resilience are widely involved [29]. Actually, these survivors have had to deal in the past with a major traumatic experience that completely changed their life, their identity, and their way of constructing themselves [30]. Thus, it is likely that anything they may experience in life other than childhood cancer seems less traumatic, developing in them a posttraumatic growth, a positive benefit, and a resilience, as important positive life changes attributable to cancer [29]. Indeed, examples of this positive effect of cancer were found in some of the free comments from CCS.

However, multivariate analysis showed that gender and socioeconomic status including living conditions significantly predicted the likelihood of reporting poorer mental health in CCS. This is consistent with the results of previous studies showing poorer mental health in women and in the most economically disadvantaged, whether in the general population or among cancer survivors [13,26,31,32,33,34,35]. Pre-existing gender differences in reported mental health would be exacerbated by the consequences of a pandemic [36,37]. Indeed, since the start of the COVID-19 pandemic, women have undergone both professional and domestic distress. It turns out that they were more likely to lose their job and to have poorer teleworking conditions than men [38]. In addition, the increased workload associated with domestic tasks and child care resulting from the closure of schools could also explain the increased risk of psychological distress among women [39,40].

This article also showed in descriptive analyses that survivors who had been diagnosed with certain types of cancer reported lower mental health scores, as was the case with brain cancer. Indeed, due to their diagnosis, the intense treatments, and the serious consequences and disabilities they have suffered, CCS of a brain tumor have been reported in several studies as having a poorer quality of life compared with survivors with other diagnoses [13,28,41]. Several factors could worsen the pre-existing mental vulnerability in CCS of a brain tumor, such as difficulties in accessing care, in particular supportive care, which would have been interrupted during the lockdown (e.g., physiotherapy, psychotherapy, etc.), or a higher perceived risk of infection, which we found, unsurprisingly, among the predictors of poor mental health in this study. Finally, CCS stating that the great lockdown had led them to remember their childhood cancer more were also more likely to report poorer mental health, with a consequent increased feeling of vulnerability. This result shows that the positive effect of the notion of childhood cancer was not shared by all respondents, hence consolidating their mixed free comments.

### Study Strengths and Limitations

In this article, we offer comparisons between CCS and the FGP in terms of mental health-related quality of life, data hitherto absent from the literature. Another strength of this study is that the FCCSS cohort brings together multiple data sources and provides ascertained information on late cardiac and endocrinological effects, among other late chronic health conditions. Despite these strengths, several limitations should be considered when interpreting the results. First, the response rate did not exceed 21%. Indeed, online surveys have been reported in the literature to be associated with a lower response rate compared with paper surveys [42]. In addition, as in any study based on self-report outcomes, there may be desirability and memory bias. This memory bias could be highlighted given the differences in the timing at which our study and that carried out in the general population were conducted. Comparisons could be limited and therefore should be cautiously interpreted. Several differences were found between respondents and non-respondents regarding individual and clinical characteristics that could skew the prevalence of the results reported above. Nevertheless, we applied in all statistical analyses weights calculated as the inverse of the probability of participating in the survey for each participant, the objective being to reduce the impact of the potential non-response bias on the etiological analyses. Finally, data comparing survivors and the general population were not collected in the same period, though in both settings, they were collected retrospectively and dealt with the same time period (i.e., psychological status during the great lockdown). Another possible limitation of this study lies in the use of the mental component of HRQoL, which is a subjective assessment of one’s health status, but it remains among the mental health indicators widely used in the field of epidemiology.

## 5. Conclusions

Given the pre-existing vulnerability of some CCS to mental distress, the psychological consequences of COVID-19 should receive attention from organizations, psychologists, and other health care providers to offer appropriate psychological support during these uncommon circumstances. In addition, identifying subgroups of CCS at higher risk for poor mental health is crucial for the implementation of targeted intervention strategies.

## Figures and Tables

**Table 1:** Comparison of demographic, socioeconomic, and clinical characteristics between respondents and non-respondents to the survey.

	Non-Respondents		Respondents		
	*n* = 2148 (79%)		*n* = 580 (21%)		
	*N*	%	*N*	%	*p*
**Age at survey (years)**					<0.0001
<39	1074	50	221	38	
40–49	720	34	222	38	
≥50	354	16	137	24	
**Gender**					0.5376
Men	1042	49	273	47	
Women	1106	51	307	53	
**Education level**					<0.0001
Less than high school	396	19	81	14	
High school graduate	758	37	154	27	
College graduate	924	44	339	59	
**Decade of diagnosis of first cancer**					<0.0001
<1970	98	5	55	9	
1970–1979	330	15	132	23	
1980–1989	792	37	186	32	
≥1990	928	43	207	36	
**Childhood cancer type**					0.0024
Nephroblastoma	329	15	109	19	
Neuroblastoma	316	15	94	16	
Lymphoma	418	19	116	20	
Soft tissue sarcoma	222	10	66	11	
Bone	226	11	59	10	
Brain tumor	245	12	32	6	
Other ^†^	392	18	104	18	
**Chemotherapy**					0.6177
No	422	21	110	20	
Yes	1630	79	451	80	
**Radiotherapy**					0.0305
No	1075	52	265	47	
Yes	977	48	296	53	
**Second cancer** ^‡^					0.2856
No	1895	92	511	91	
Yes	158	8	51	9	
**Diabetes** ^‡^					0.0065
No	2097	98	554	96	
Yes	51	2	26	4	
**Cardiac disease** ^‡^					0.0001
No	2028	95	522	90	
Grades 1 and 2	42	2	25	4	
Grades ≥ 3	69	3	32	6	

^†^ Other: carcinomas, gonadal tumors, thyroid, retinoblastoma; ^‡^ Ascertained from physicians’ reports and complemented by data extracted from patients’ medical records and health insurance databases.

**Table 2:** Participants′ Mental Health Inventory-5 (MHI-5) scores compared with those of the general population using Z-scores matched for age and gender.

**MHI-5 Scores**					
**Overall MHI-5 score**		**By gender**			
**All (*n* = 505)**		**Men (*n* = 238)**		**Women (*n* = 267)**	
Mean Z-score (95% CI)		Mean Z-score (95% CI)		Mean Z-score (95% CI)	
−0.01 (−0.10 to 0.08)		0.11 (−0.02 to 0.23)		−0.11 (−0.24 to 0.02)	
**By educational level**					
**Less than high school (*n* = 62)**		**High school graduate (*n* = 127)**		**College graduate (*n* = 316)**	
Mean Z-score (95% CI)		Mean Z-score (95% CI)		Mean Z-score (95% CI)	
0.15 (−0.10 to 0.39)		−0.16 (−0.36 to 0.04)		0.04 (−0.07 to 0.15)	
**By second cancer**					
**No (*n* = 440)**			**Yes (*n* = 47)**		
Mean Z-score (95% CI)			Mean Z-score (95% CI)		
−0.02 (−0.11 to 0.08)			0.04 (−0.29 to 0.37)		
**By cardiac disease**					
**No (*n* = 453)**			**Yes (*n* = 52)**		
Mean Z-score (95% CI)			Mean Z-score (95% CI)		
−0.00 (−0.10 to 0.09)			−0.05 (−0.37 to 0.26)		

CI, confidence interval.

**Table 3:** Variables and factors associated with the Mental Health Inventory-5 (MHI-5) score.

	**Univariate Analyses**		**Multivariate Analyses** **(*n* = 497)**	
	Unadjusted β and 95% CI	*p*	Adjusted β and 95% CI	*p*
**Age at the survey (years)**	0.12 [0.04 to 0.21]	0.0037	0.10 [0.01 to 0.19]	0.0315
**Gender**				
Women	−4.76 [−6.40 to −3.11]	<0.0001	−3.55 [−5.20 to −1.89]	<0.0001
**Decade of diagnosis of first cancer**				
≥1990 versus earlier	−2.33 [−4.02 to −0.64]	0.0071		
**Education level**				
Less than high school	1.84 [−0.40 to 4.07]	0.1066	-	
High school graduate	−2.58 [−4.35 to −0.82]	0.0042	−1.18 [−2.89 to 0.53]	0.1761
College graduate	1.29 [−0.40 to 2.97]	0.1342	-	
**Chemotherapy**	0.84 [−1.35 to 3.03]	0.4498	2.35 [0.25 to 4.46]	0.0287
**Radiotherapy**	0.90 [−0.82 to 2.63]	0.3047	0.20 [−1.51 to 1.90]	0.8190
**Second cancer**	0.60 [−2.46 to 3.66]	0.7002	−0.64 [−3.52 to 2.25]	0.6643
**Cardiac disease**	−0.38 [−3.32 to 2.57]	0.8015	0.39 [−2.39 to 3.16]	0.7847
**Having a relative who had been hospitalized or had died following COVID-19**	−3.34 [−5.57 to −1.10]	0.0035	−2.70 [−4.81 to −0.58]	0.0126
**Change of occupational situation during the great lockdown**				
Termination of the contract by the employer	−9.61 [−15.12 to −4.10]	0.0007	−7.64 [−12.87 to −2.42]	0.0042
Stop or significant decrease in professional activity (self-employed)	−5.60 [−9.60 to −1.61]	0.0061	−7.50 [−11.13 to −3.86]	<0.0001
**Fear that health might be endangered by the working conditions due to the COVID-19 pandemic**	−2.77 [−4.97 to −0.57]	0.0137	−1.44 [−3.55 to 0.67]	0.1809
**Forgoing health care since the start of the pandemic**	−3.08 [−6.01 to −0.99]	0.0039		
**Remembering childhood cancer more during the great lockdown**	−3.82 [−5.59 to −2.05]	<0.0001	−2.45 [−4.20 to −0.70]	0.0061
**Number of rooms in the house**	1.15 [0.71 to 1.59]	<0.0001	0.47 [−0.04 to 0.98]	0.0728
**Presence of a garden in the house**	4.47 [2.77 to 6.16]	<0.0001	2.58 [0.55 to 4.61]	0.0127
**Perceived infection risk**	−0.95 [−1.24 to −0.66]	<0.0001	−0.68 [−0.98 to −0.37]	<0.0001

CI, confidence interval.

## Data Availability

The data that support the findings of this study are available on request from the corresponding author.
